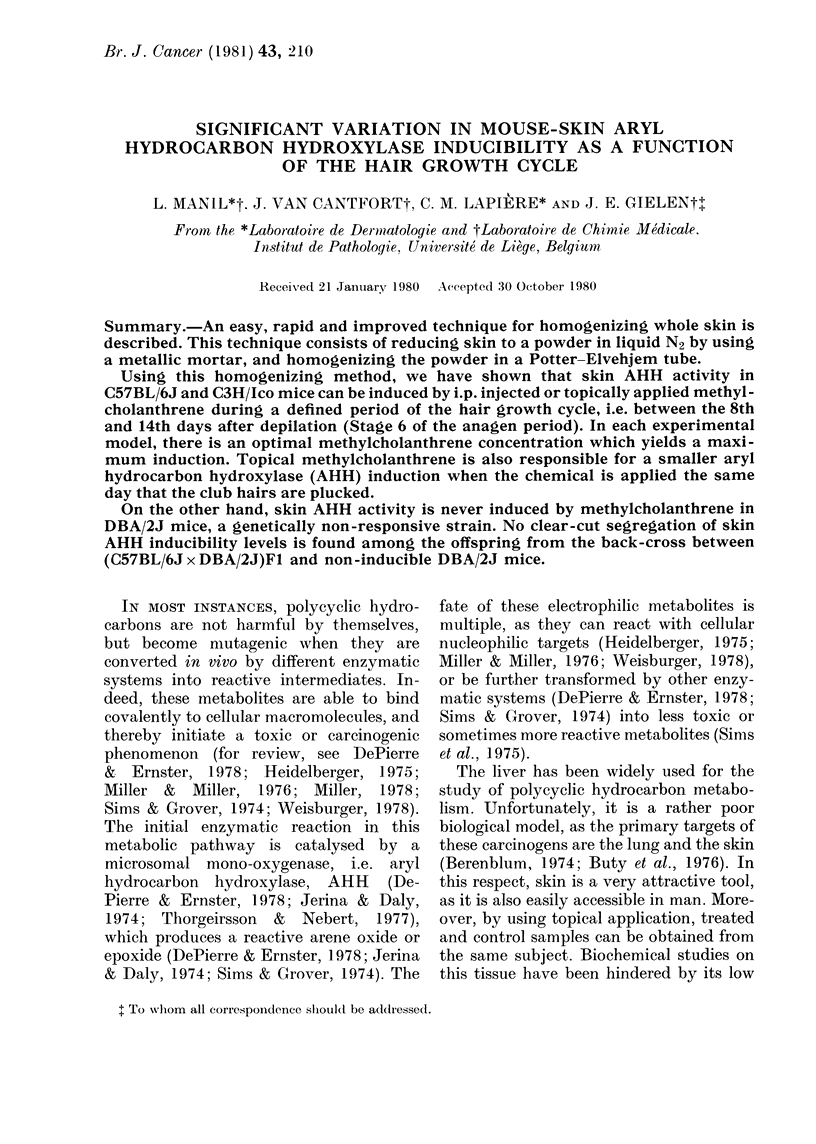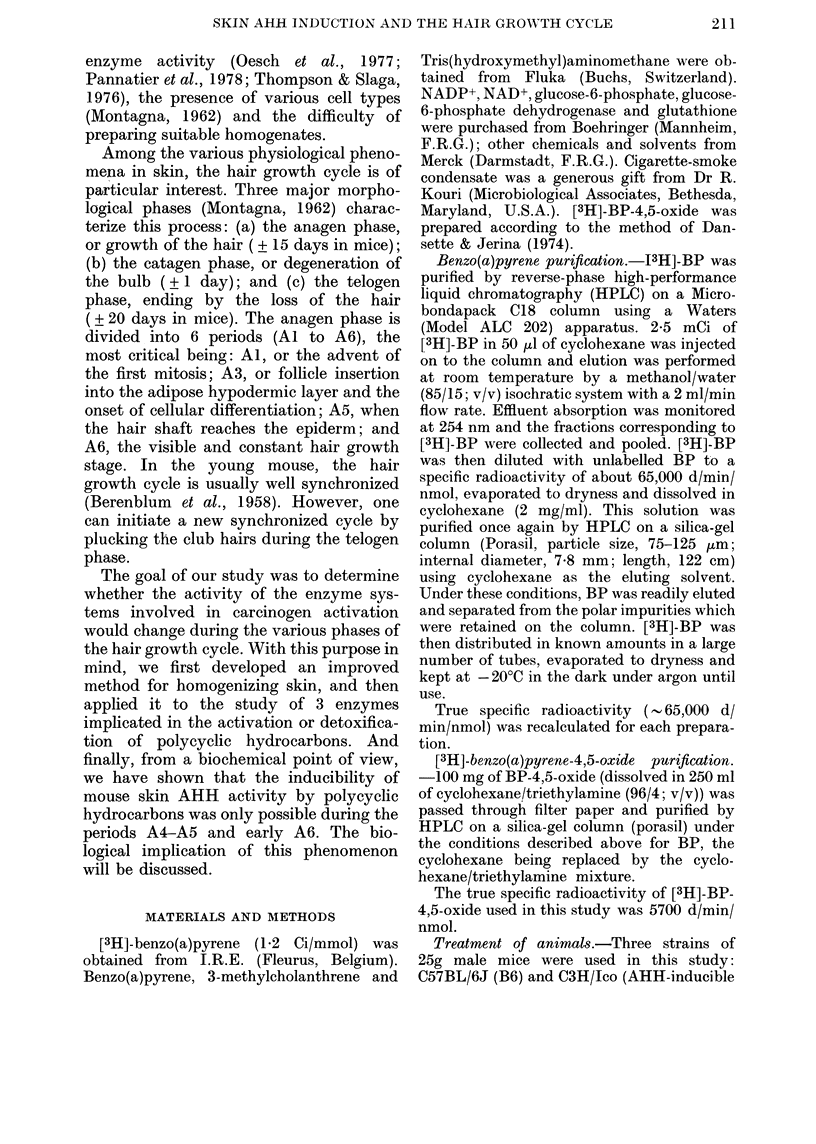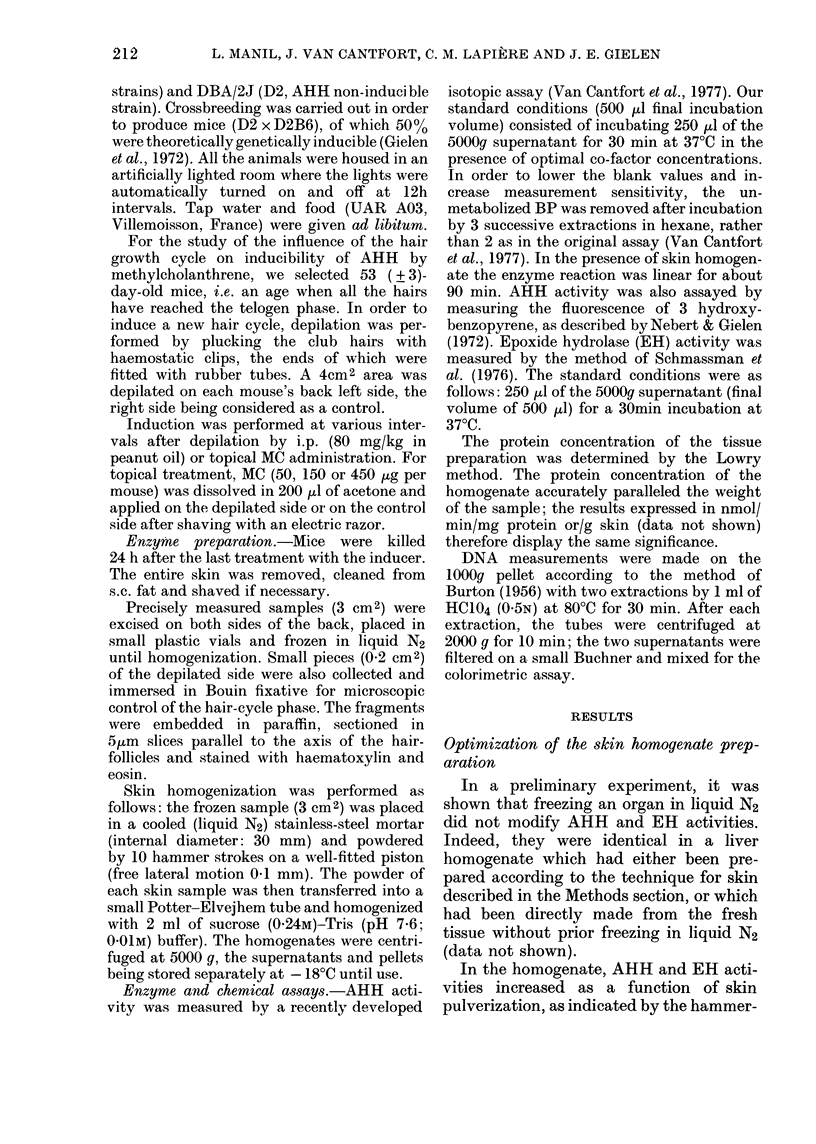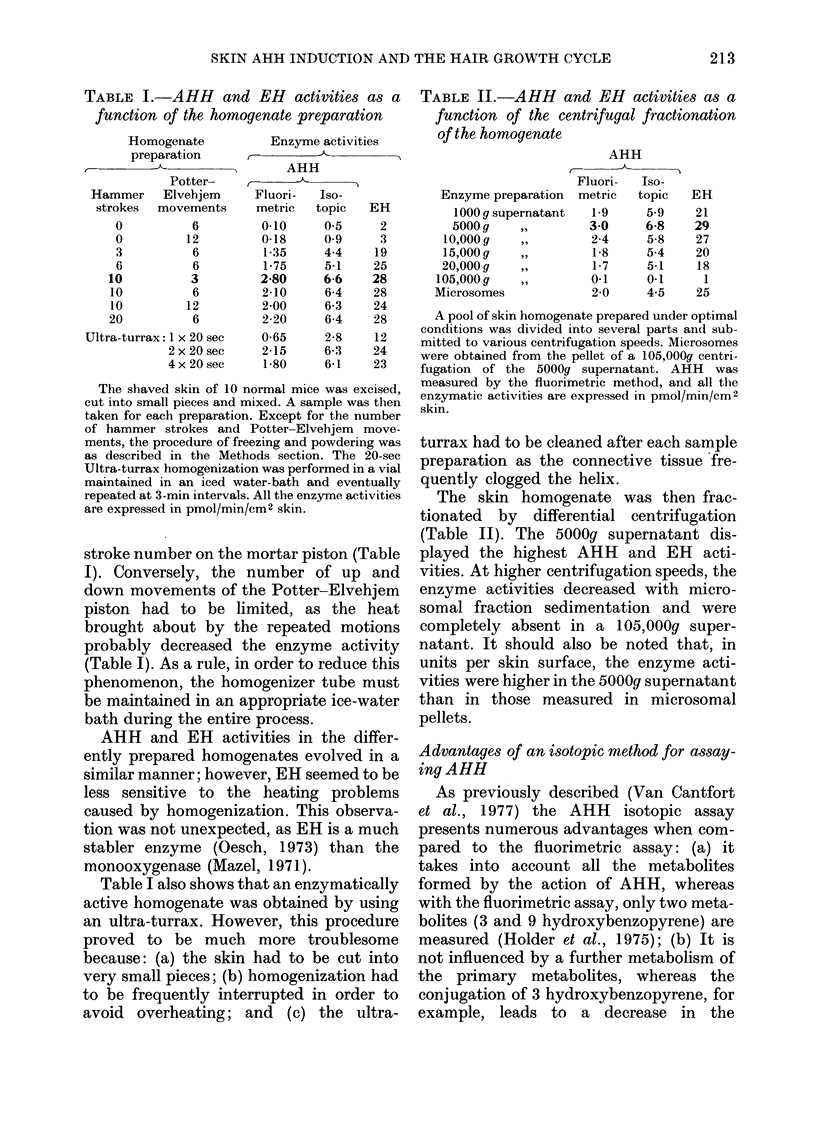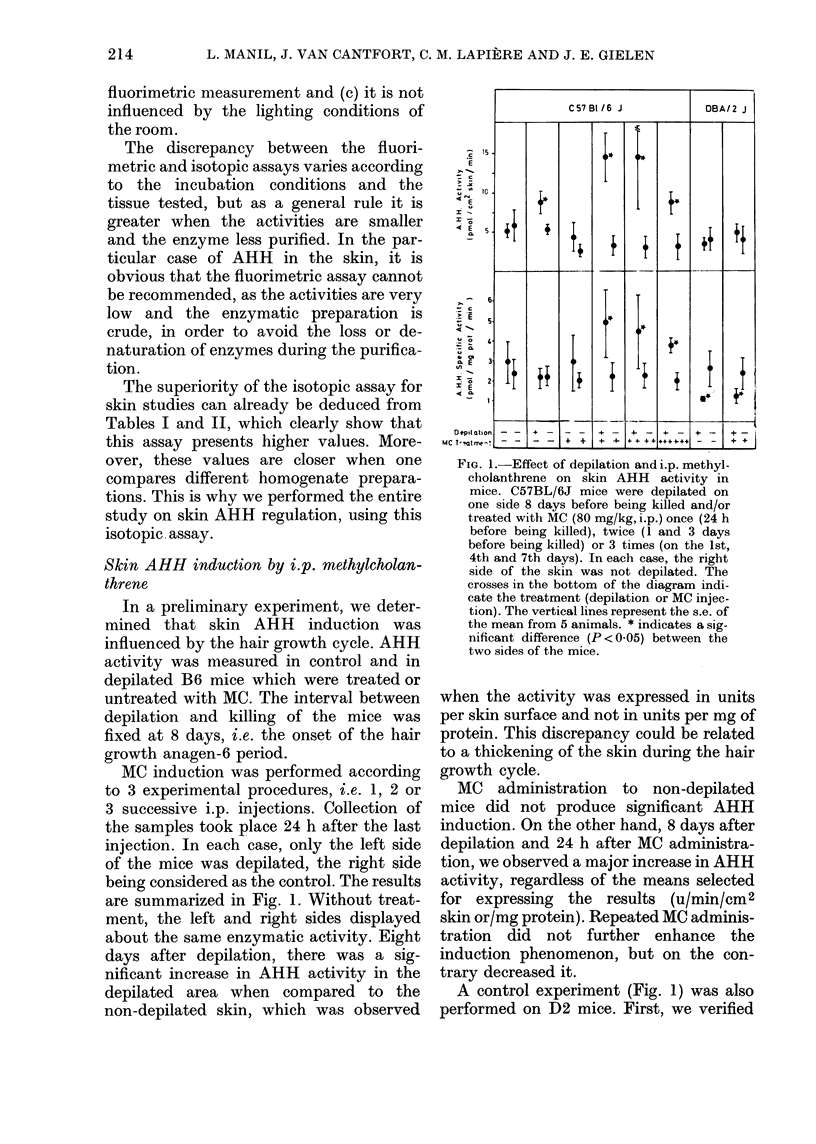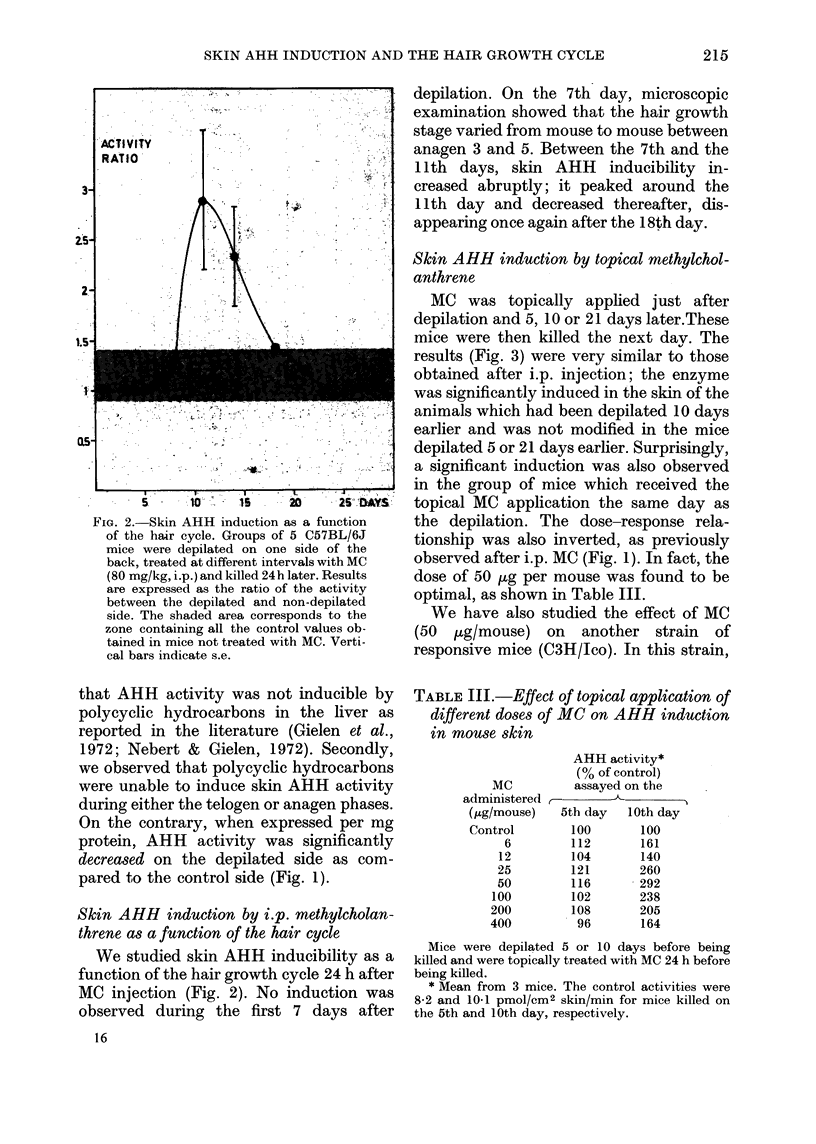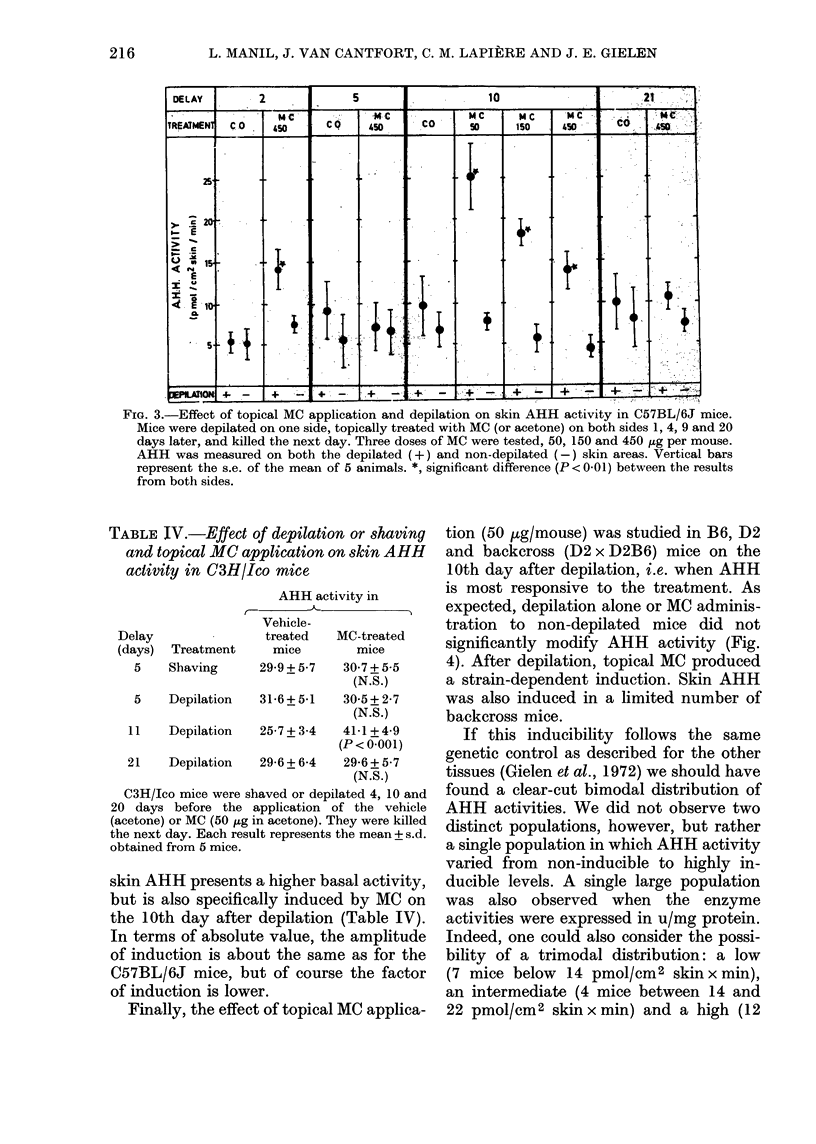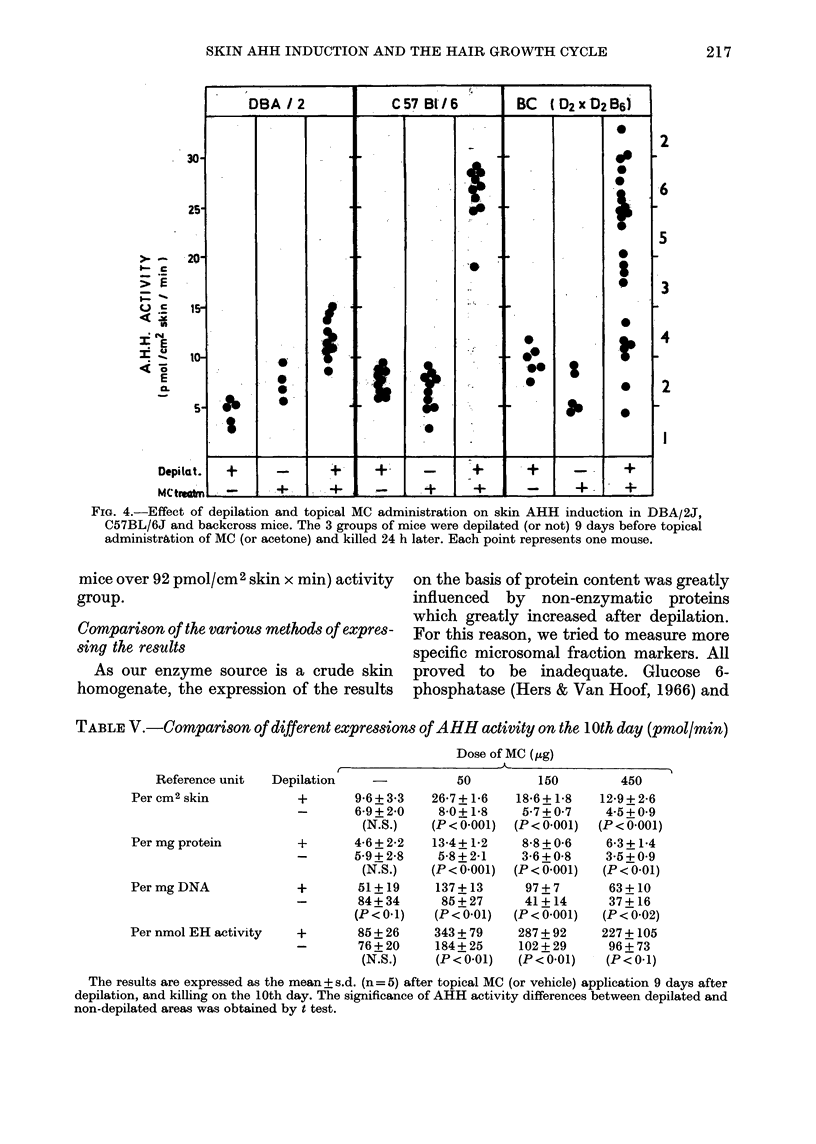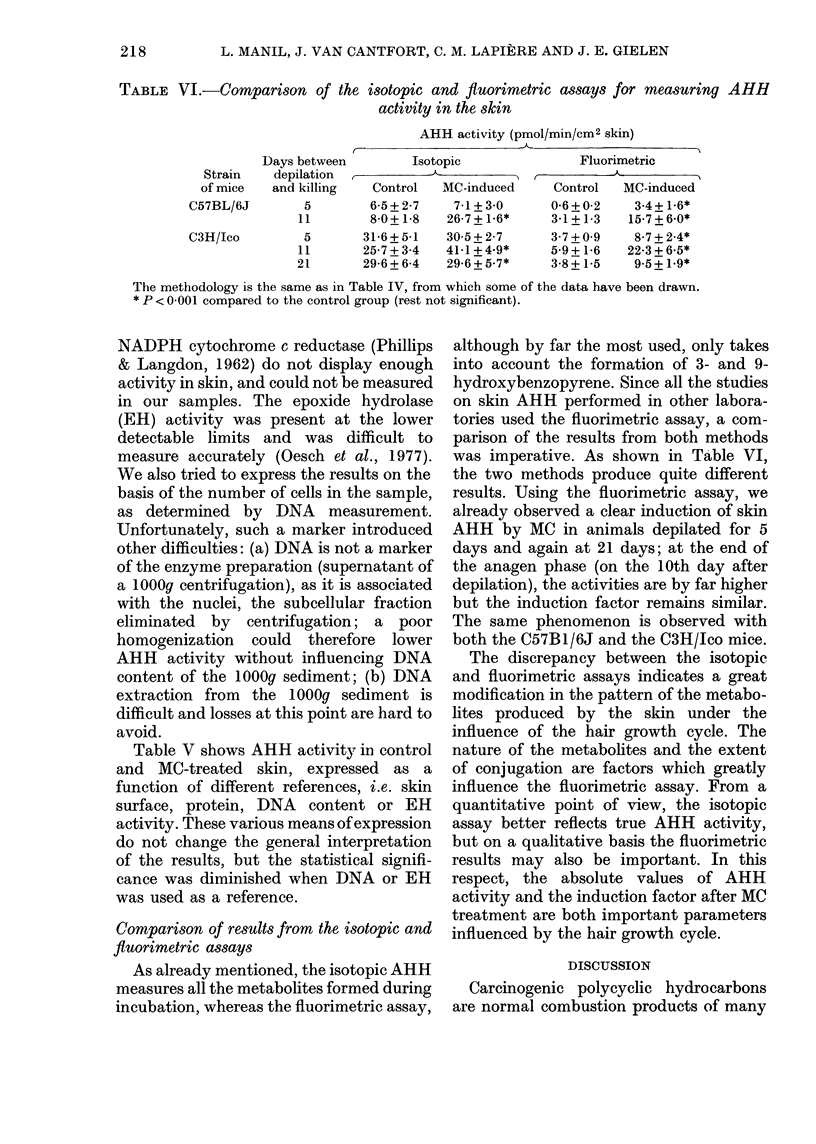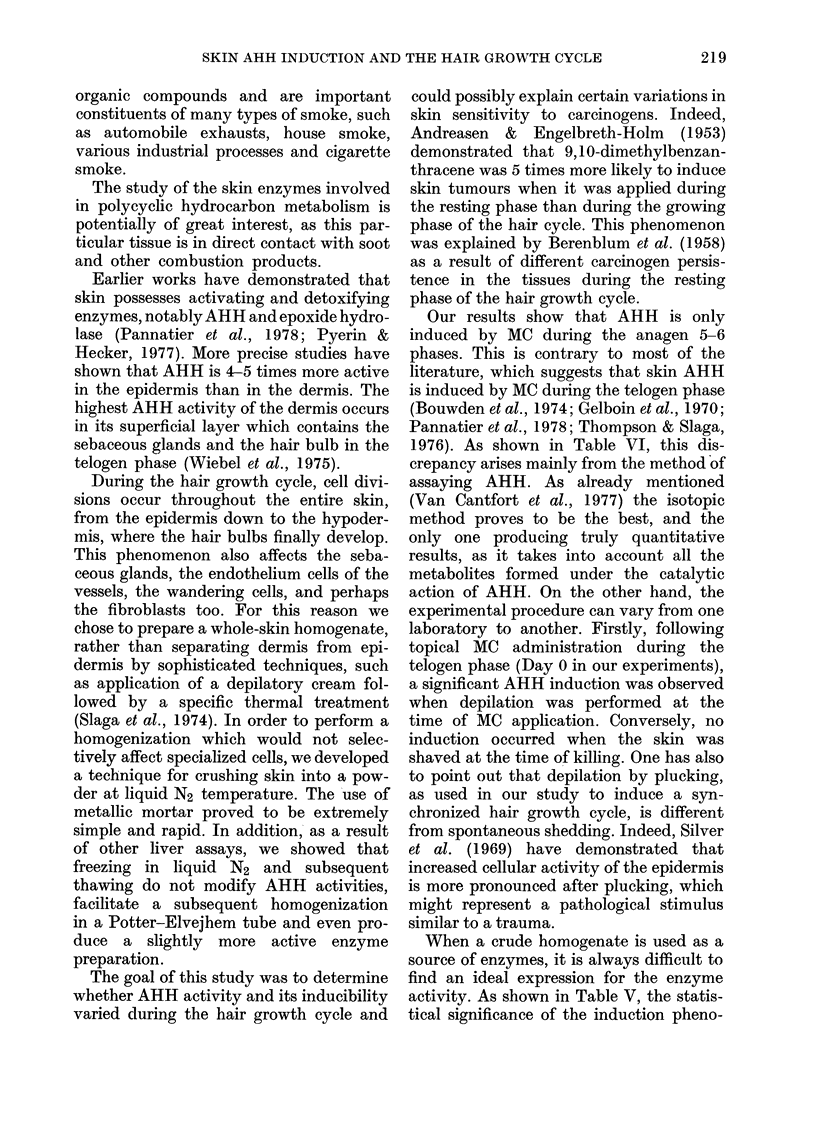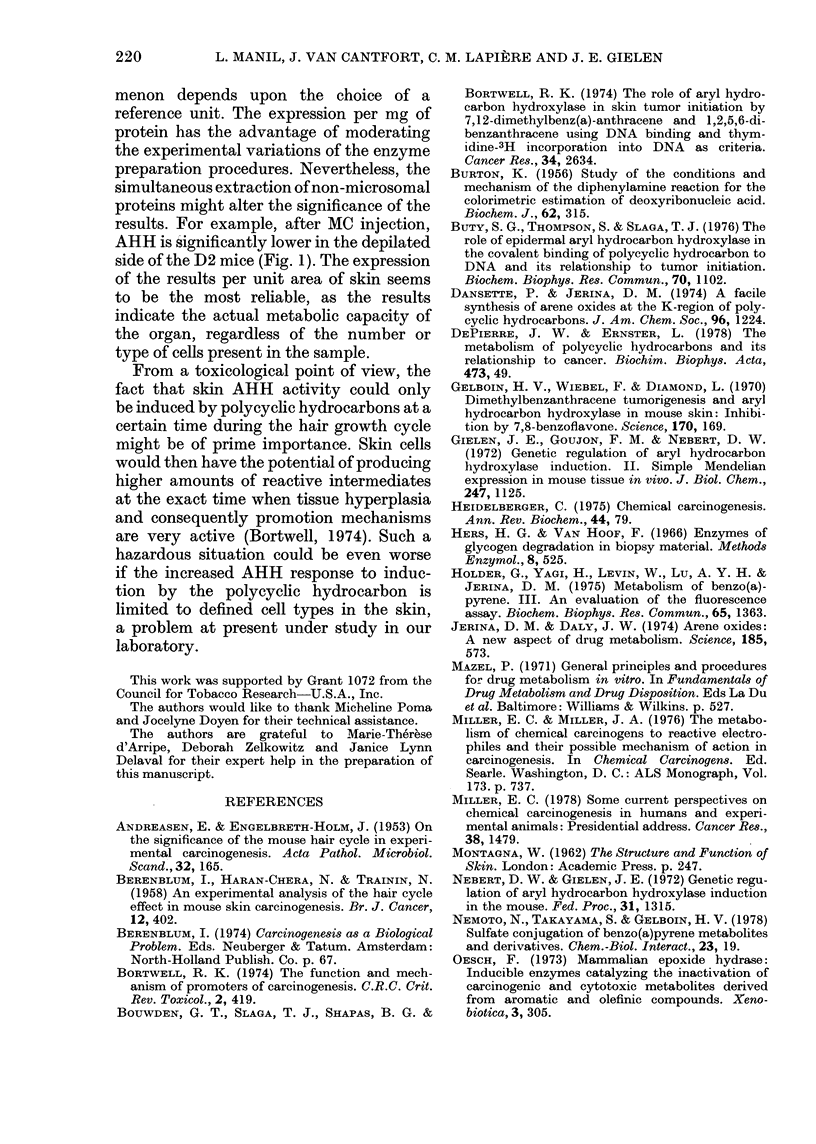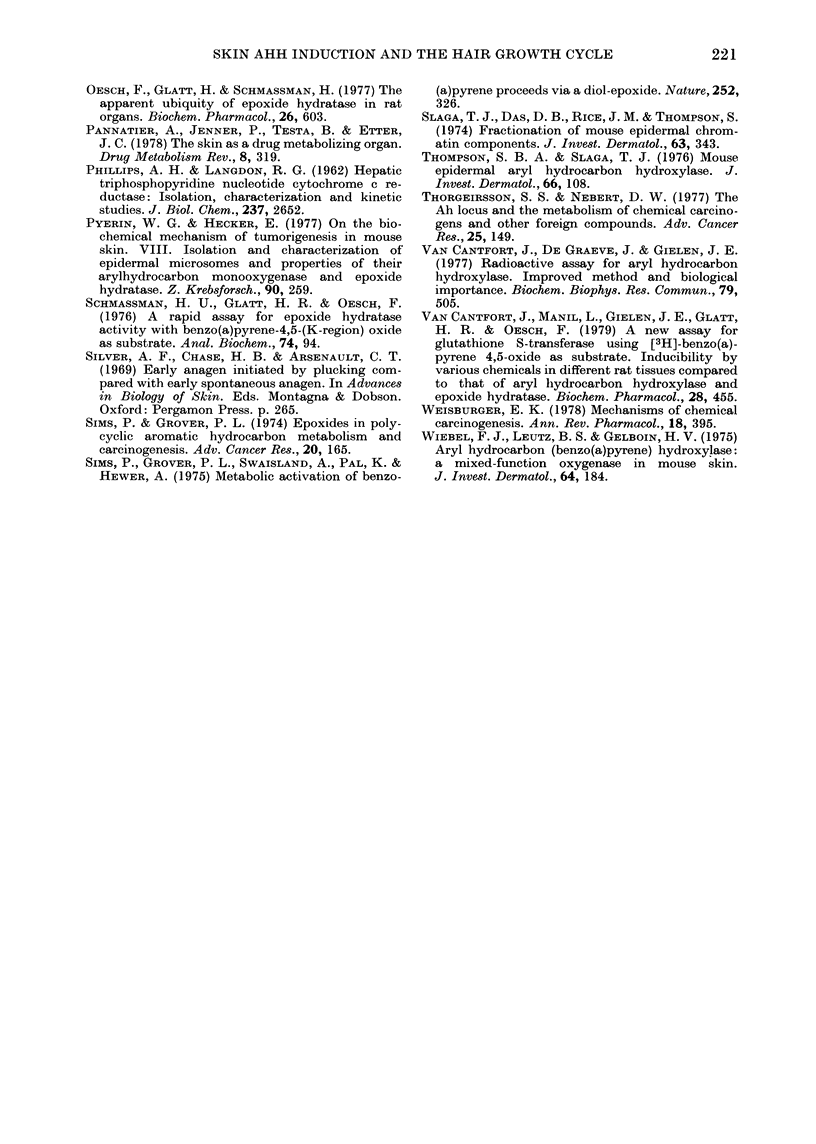# Significant variation in mouse-skin aryl hydrocarbon hydroxylase inducibility as a function of the hair growth cycle.

**DOI:** 10.1038/bjc.1981.30

**Published:** 1981-02

**Authors:** L. Manil, J. Van Cantfort, C. M. Lapière, J. E. Gielen

## Abstract

An easy, rapid and improved technique for homogenizing whole skin is described. This technique consists of reducing skin to a powder in liquid N2 by using a metallic mortar, and homogenizing the powder in a Potter-Elvehjem tube. Using this homogenizing method, we have shown that skin AHH activity in C57BL/6K and C3H/Ico mice can be induced by i.p. injected or topically applied methylcholanthrene during a defined period of the hair growth cycle, i.e. between the 8th and 14th days after depilation (Stage 6 of the anagen period). In each experimental model, there is an optimal methylcholanthrene concentration which yields a maximum induction. Topical methylcholanthrene is also responsible for a smaller aryl hydrocarbon hydroxylase (AHH) induction when the chemical is applied the same day that the club hairs are plucked. On the other hand, skin AHH activity is never induced by methylcholanthrene in DBA/2J mice, a genetically non-responsive strain. No clear-cut segregation of skin AHH inducibility levels is found among the offspring from the back-cross between (C57BL/6J X DBA/2J)F1 and non-inducible DBA/2J mice.


*Br. J. Cancer* (1981) **43**, 210

### SIGNIFICANT VARIATION IN MOUSE-SKIN ARYL HYDROCARBON HYDROXYLASE INDUCIBILITY AS A FUNCTION OF THE HAIR GROWTH CYCLE

L. MANIL*†, J. VAN CANTFORT†, C. M. LAPIÈRE* AND J. E. GIELEN†‡

*From the *Laboratoire de Dermatologie and †Laboratoire de Chimie Médicale, Institut de Pathologie, Université de Liège, Belgium*

Received 21 January 1980 Accepted 30 October 1980

**Summary.—An easy, rapid and improved technique for homogenizing whole skin is described. This technique consists of reducing skin to a powder in liquid $N_2$ by using a metallic mortar, and homogenizing the powder in a Potter–Elvehjem tube.**

**Using this homogenizing method, we have shown that skin AHH activity in C57BL/6J and C3H/Ico mice can be induced by i.p. injected or topically applied methylcholanthrene during a defined period of the hair growth cycle, i.e. between the 8th and 14th days after depilation (Stage 6 of the anagen period). In each experimental model, there is an optimal methylcholanthrene concentration which yields a maximum induction. Topical methylcholanthrene is also responsible for a smaller aryl hydrocarbon hydroxylase (AHH) induction when the chemical is applied the same day that the club hairs are plucked.**

**On the other hand, skin AHH activity is never induced by methylcholanthrene in DBA/2J mice, a genetically non-responsive strain. No clear-cut segregation of skin AHH inducibility levels is found among the offspring from the back-cross between (C57BL/6J × DBA/2J)F1 and non-inducible DBA/2J mice.**

IN MOST INSTANCES, polycyclic hydrocarbons are not harmful by themselves, but become mutagenic when they are converted *in vivo* by different enzymatic systems into reactive intermediates. Indeed, these metabolites are able to bind covalently to cellular macromolecules, and thereby initiate a toxic or carcinogenic phenomenon (for review, see DePierre & Ernster, 1978; Heidelberger, 1975; Miller & Miller, 1976; Miller, 1978; Sims & Grover, 1974; Weisburger, 1978). The initial enzymatic reaction in this metabolic pathway is catalysed by a microsomal mono-oxygenase, i.e. aryl hydrocarbon hydroxylase, AHH (DePierre & Ernster, 1978; Jerina & Daly, 1974; Thorgeirsson & Nebert, 1977), which produces a reactive arene oxide or epoxide (DePierre & Ernster, 1978; Jerina & Daly, 1974; Sims & Grover, 1974). The fate of these electrophilic metabolites is multiple, as they can react with cellular nucleophilic targets (Heidelberger, 1975; Miller & Miller, 1976; Weisburger, 1978), or be further transformed by other enzymatic systems (DePierre & Ernster, 1978; Sims & Grover, 1974) into less toxic or sometimes more reactive metabolites (Sims *et al.*, 1975).

The liver has been widely used for the study of polycyclic hydrocarbon metabolism. Unfortunately, it is a rather poor biological model, as the primary targets of these carcinogens are the lung and the skin (Berenblum, 1974; Buty *et al.*, 1976). In this respect, skin is a very attractive tool, as it is also easily accessible in man. Moreover, by using topical application, treated and control samples can be obtained from the same subject. Biochemical studies on this tissue have been hindered by its low

‡ To whom all correspondence should be addressed.

enzyme activity (Oesch *et al.*, 1977; Pannatier *et al.*, 1978; Thompson & Slaga, 1976), the presence of various cell types (Montagna, 1962) and the difficulty of preparing suitable homogenates.

Among the various physiological phenomena in skin, the hair growth cycle is of particular interest. Three major morphological phases (Montagna, 1962) characterize this process: (a) the anagen phase, or growth of the hair ($\pm$15 days in mice); (b) the catagen phase, or degeneration of the bulb ($\pm$1 day); and (c) the telogen phase, ending by the loss of the hair ($\pm$20 days in mice). The anagen phase is divided into 6 periods (A1 to A6), the most critical being: A1, or the advent of the first mitosis; A3, or follicle insertion into the adipose hypodermic layer and the onset of cellular differentiation; A5, when the hair shaft reaches the epiderm; and A6, the visible and constant hair growth stage. In the young mouse, the hair growth cycle is usually well synchronized (Berenblum *et al.*, 1958). However, one can initiate a new synchronized cycle by plucking the club hairs during the telogen phase.

The goal of our study was to determine whether the activity of the enzyme systems involved in carcinogen activation would change during the various phases of the hair growth cycle. With this purpose in mind, we first developed an improved method for homogenizing skin, and then applied it to the study of 3 enzymes implicated in the activation or detoxification of polycyclic hydrocarbons. And finally, from a biochemical point of view, we have shown that the inducibility of mouse skin AHH activity by polycyclic hydrocarbons was only possible during the periods A4–A5 and early A6. The biological implication of this phenomenon will be discussed.

### MATERIALS AND METHODS

[$^3$H]-benzo(a)pyrene (1·2 Ci/mmol) was obtained from I.R.E. (Fleurus, Belgium). Benzo(a)pyrene, 3-methylcholanthrene and Tris(hydroxymethyl)aminomethane were obtained from Fluka (Buchs, Switzerland). $NADP^+$, $NAD^+$, glucose-6-phosphate, glucose-6-phosphate dehydrogenase and glutathione were purchased from Boehringer (Mannheim, F.R.G.); other chemicals and solvents from Merck (Darmstadt, F.R.G.). Cigarette-smoke condensate was a generous gift from Dr R. Kouri (Microbiological Associates, Bethesda, Maryland, U.S.A.). [$^3$H]-BP-4,5-oxide was prepared according to the method of Dansette & Jerina (1974).

*Benzo(a)pyrene purification.*—I$^3$H]-BP was purified by reverse-phase high-performance liquid chromatography (HPLC) on a Microbondapack C18 column using a Waters (Model ALC 202) apparatus. 2·5 mCi of [$^3$H]-BP in 50 $\mu$l of cyclohexane was injected on to the column and elution was performed at room temperature by a methanol/water (85/15; v/v) isochratic system with a 2 ml/min flow rate. Effluent absorption was monitored at 254 nm and the fractions corresponding to [$^3$H]-BP were collected and pooled. [$^3$H]-BP was then diluted with unlabelled BP to a specific radioactivity of about 65,000 d/min/nmol, evaporated to dryness and dissolved in cyclohexane (2 mg/ml). This solution was purified once again by HPLC on a silica-gel column (Porasil, particle size, 75–125 $\mu$m; internal diameter, 7·8 mm; length, 122 cm) using cyclohexane as the eluting solvent. Under these conditions, BP was readily eluted and separated from the polar impurities which were retained on the column. [$^3$H]-BP was then distributed in known amounts in a large number of tubes, evaporated to dryness and kept at $-20$°C in the dark under argon until use.

True specific radioactivity ($\sim$65,000 d/min/nmol) was recalculated for each preparation.

[$^3$H]-*benzo(a)pyrene*-4,5-*oxide purification.*—100 mg of BP-4,5-oxide (dissolved in 250 ml of cyclohexane/triethylamine (96/4; v/v)) was passed through filter paper and purified by HPLC on a silica-gel column (porasil) under the conditions described above for BP, the cyclohexane being replaced by the cyclohexane/triethylamine mixture.

The true specific radioactivity of [$^3$H]-BP-4,5-oxide used in this study was 5700 d/min/nmol.

*Treatment of animals.*—Three strains of 25g male mice were used in this study: C57BL/6J (B6) and C3H/Ico (AHH-inducible strains) and DBA/2J (D2, AHH non-inducible strain). Crossbreeding was carried out in order to produce mice (D2 × D2B6), of which 50% were theoretically genetically inducible (Gielen *et al.*, 1972). All the animals were housed in an artificially lighted room where the lights were automatically turned on and off at 12h intervals. Tap water and food (UAR A03, Villemoisson, France) were given *ad libitum.*

For the study of the influence of the hair growth cycle on inducibility of AHH by methylcholanthrene, we selected 53 (±3)-day-old mice, *i.e.* an age when all the hairs have reached the telogen phase. In order to induce a new hair cycle, depilation was performed by plucking the club hairs with haemostatic clips, the ends of which were fitted with rubber tubes. A 4cm$^2$ area was depilated on each mouse's back left side, the right side being considered as a control.

Induction was performed at various intervals after depilation by i.p. (80 mg/kg in peanut oil) or topical MC administration. For topical treatment, MC (50, 150 or 450 $\mu$g per mouse) was dissolved in 200 $\mu$l of acetone and applied on the depilated side or on the control side after shaving with an electric razor.

*Enzyme preparation.*—Mice were killed 24 h after the last treatment with the inducer. The entire skin was removed, cleaned from s.c. fat and shaved if necessary.

Precisely measured samples (3 cm$^2$) were excised on both sides of the back, placed in small plastic vials and frozen in liquid $N_2$ until homogenization. Small pieces (0·2 cm$^2$) of the depilated side were also collected and immersed in Bouin fixative for microscopic control of the hair-cycle phase. The fragments were embedded in paraffin, sectioned in 5$\mu$m slices parallel to the axis of the hair-follicles and stained with haematoxylin and eosin.

Skin homogenization was performed as follows: the frozen sample (3 cm$^2$) was placed in a cooled (liquid $N_2$) stainless-steel mortar (internal diameter: 30 mm) and powdered by 10 hammer strokes on a well-fitted piston (free lateral motion 0·1 mm). The powder of each skin sample was then transferred into a small Potter–Elvejhem tube and homogenized with 2 ml of sucrose (0·24M)–Tris (pH 7·6; 0·01M) buffer). The homogenates were centrifuged at 5000 *g*, the supernatants and pellets being stored separately at −18°C until use.

*Enzyme and chemical assays.*—AHH activity was measured by a recently developed isotopic assay (Van Cantfort *et al.*, 1977). Our standard conditions (500 $\mu$l final incubation volume) consisted of incubating 250 $\mu$l of the 5000*g* supernatant for 30 min at 37°C in the presence of optimal co-factor concentrations. In order to lower the blank values and increase measurement sensitivity, the unmetabolized BP was removed after incubation by 3 successive extractions in hexane, rather than 2 as in the original assay (Van Cantfort *et al.*, 1977). In the presence of skin homogenate the enzyme reaction was linear for about 90 min. AHH activity was also assayed by measuring the fluorescence of 3 hydroxybenzopyrene, as described by Nebert & Gielen (1972). Epoxide hydrolase (EH) activity was measured by the method of Schmassman *et al.* (1976). The standard conditions were as follows: 250 $\mu$l of the 5000*g* supernatant (final volume of 500 $\mu$l) for a 30min incubation at 37°C.

The protein concentration of the tissue preparation was determined by the Lowry method. The protein concentration of the homogenate accurately paralleled the weight of the sample; the results expressed in nmol/min/mg protein or/g skin (data not shown) therefore display the same significance.

DNA measurements were made on the 1000*g* pellet according to the method of Burton (1956) with two extractions by 1 ml of $HClO_4$ (0·5N) at 80°C for 30 min. After each extraction, the tubes were centrifuged at 2000 *g* for 10 min; the two supernatants were filtered on a small Buchner and mixed for the colorimetric assay.

### RESULTS

### *Optimization of the skin homogenate preparation*

In a preliminary experiment, it was shown that freezing an organ in liquid $N_2$ did not modify AHH and EH activities. Indeed, they were identical in a liver homogenate which had either been prepared according to the technique for skin described in the Methods section, or which had been directly made from the fresh tissue without prior freezing in liquid $N_2$ (data not shown).

In the homogenate, AHH and EH activities increased as a function of skin pulverization, as indicated by the hammer-

TABLE I.—*AHH and EH activities as a function of the homogenate preparation*

| Homogenate preparation | | Enzyme activities | | |
|---|---|---|---|---|
| | | AHH | | |
| Hammer strokes | Potter–Elvehjem movements | Fluorimetric | Isotopic | EH |
| 0 | 6 | 0·10 | 0·5 | 2 |
| 0 | 12 | 0·18 | 0·9 | 3 |
| 3 | 6 | 1·35 | 4·4 | 19 |
| 6 | 6 | 1·75 | 5·1 | 25 |
| **10** | **3** | **2·80** | **6·6** | **28** |
| 10 | 6 | 2·10 | 6·4 | 28 |
| 10 | 12 | 2·00 | 6·3 | 24 |
| 20 | 6 | 2·20 | 6·4 | 28 |
| Ultra-turrax: | 1 x 20 sec | 0·65 | 2·8 | 12 |
| | 2 x 20 sec | 2·15 | 6·3 | 24 |
| | 4 x 20 sec | 1·80 | 6·1 | 23 |

The shaved skin of 10 normal mice was excised, cut into small pieces and mixed. A sample was then taken for each preparation. Except for the number of hammer strokes and Potter–Elvehjem movements, the procedure of freezing and powdering was as described in the Methods section. The 20-sec Ultra-turrax homogenization was performed in a vial maintained in an iced water-bath and eventually repeated at 3-min intervals. All the enzyme activities are expressed in pmol/min/cm$^2$ skin.

stroke number on the mortar piston (Table I). Conversely, the number of up and down movements of the Potter–Elvehjem piston had to be limited, as the heat brought about by the repeated motions probably decreased the enzyme activity (Table I). As a rule, in order to reduce this phenomenon, the homogenizer tube must be maintained in an appropriate ice-water bath during the entire process.

AHH and EH activities in the differently prepared homogenates evolved in a similar manner; however, EH seemed to be less sensitive to the heating problems caused by homogenization. This observation was not unexpected, as EH is a much stabler enzyme (Oesch, 1973) than the monooxygenase (Mazel, 1971).

Table I also shows that an enzymatically active homogenate was obtained by using an ultra-turrax. However, this procedure proved to be much more troublesome because: (a) the skin had to be cut into very small pieces; (b) homogenization had to be frequently interrupted in order to avoid overheating; and (c) the ultra-

TABLE II.—*AHH and EH activities as a function of the centrifugal fractionation of the homogenate*

| | AHH | | |
|---|---|---|---|
| Enzyme preparation | Fluorimetric | Isotopic | EH |
| 1000 *g* supernatant | 1·9 | 5·9 | 21 |
| 5000 *g* ,, | **3·0** | **6·8** | **29** |
| 10,000 *g* ,, | 2·4 | 5·8 | 27 |
| 15,000 *g* ,, | 1·8 | 5·4 | 20 |
| 20,000 *g* ,, | 1·7 | 5·1 | 18 |
| 105,000 *g* ,, | 0·1 | 0·1 | 1 |
| Microsomes | 2·0 | 4·5 | 25 |

A pool of skin homogenate prepared under optimal conditions was divided into several parts and submitted to various centrifugation speeds. Microsomes were obtained from the pellet of a 105,000*g* centrifugation of the 5000*g* supernatant. AHH was measured by the fluorimetric method, and all the enzymatic activities are expressed in pmol/min/cm$^2$ skin.

turrax had to be cleaned after each sample preparation as the connective tissue frequently clogged the helix.

The skin homogenate was then fractionated by differential centrifugation (Table II). The 5000*g* supernatant displayed the highest AHH and EH activities. At higher centrifugation speeds, the enzyme activities decreased with microsomal fraction sedimentation and were completely absent in a 105,000*g* supernatant. It should also be noted that, in units per skin surface, the enzyme activities were higher in the 5000*g* supernatant than in those measured in microsomal pellets.

### *Advantages of an isotopic method for assaying AHH*

As previously described (Van Cantfort *et al.*, 1977) the AHH isotopic assay presents numerous advantages when compared to the fluorimetric assay: (a) it takes into account all the metabolites formed by the action of AHH, whereas with the fluorimetric assay, only two metabolites (3 and 9 hydroxybenzopyrene) are measured (Holder *et al.*, 1975); (b) It is not influenced by a further metabolism of the primary metabolites, whereas the conjugation of 3 hydroxybenzopyrene, for example, leads to a decrease in the fluorimetric measurement and (c) it is not influenced by the lighting conditions of the room.

The discrepancy between the fluorimetric and isotopic assays varies according to the incubation conditions and the tissue tested, but as a general rule it is greater when the activities are smaller and the enzyme less purified. In the particular case of AHH in the skin, it is obvious that the fluorimetric assay cannot be recommended, as the activities are very low and the enzymatic preparation is crude, in order to avoid the loss or denaturation of enzymes during the purification.

The superiority of the isotopic assay for skin studies can already be deduced from Tables I and II, which clearly show that this assay presents higher values. Moreover, these values are closer when one compares different homogenate preparations. This is why we performed the entire study on skin AHH regulation, using this isotopic assay.

*Skin AHH induction by i.p. methylcholanthrene*

In a preliminary experiment, we determined that skin AHH induction was influenced by the hair growth cycle. AHH activity was measured in control and in depilated B6 mice which were treated or untreated with MC. The interval between depilation and killing of the mice was fixed at 8 days, *i.e.* the onset of the hair growth anagen-6 period.

MC induction was performed according to 3 experimental procedures, *i.e.* 1, 2 or 3 successive i.p. injections. Collection of the samples took place 24 h after the last injection. In each case, only the left side of the mice was depilated, the right side being considered as the control. The results are summarized in Fig. 1. Without treatment, the left and right sides displayed about the same enzymatic activity. Eight days after depilation, there was a significant increase in AHH activity in the depilated area when compared to the non-depilated skin, which was observed

FIG. 1.—Effect of depilation and i.p. methylcholanthrene on skin AHH activity in mice. C57BL/6J mice were depilated on one side 8 days before being killed and/or treated with MC (80 mg/kg, i.p.) once (24 h before being killed), twice (1 and 3 days before being killed) or 3 times (on the 1st, 4th and 7th days). In each case, the right side of the skin was not depilated. The crosses in the bottom of the diagram indicate the treatment (depilation or MC injection). The vertical lines represent the s.e. of the mean from 5 animals. * indicates a significant difference ($P < 0{\cdot}05$) between the two sides of the mice.

when the activity was expressed in units per skin surface and not in units per mg of protein. This discrepancy could be related to a thickening of the skin during the hair growth cycle.

MC administration to non-depilated mice did not produce significant AHH induction. On the other hand, 8 days after depilation and 24 h after MC administration, we observed a major increase in AHH activity, regardless of the means selected for expressing the results (u/min/cm$^2$ skin or/mg protein). Repeated MC administration did not further enhance the induction phenomenon, but on the contrary decreased it.

A control experiment (Fig. 1) was also performed on D2 mice. First, we verified

FIG. 2.—Skin AHH induction as a function of the hair cycle. Groups of 5 C57BL/6J mice were depilated on one side of the back, treated at different intervals with MC (80 mg/kg, i.p.) and killed 24 h later. Results are expressed as the ratio of the activity between the depilated and non-depilated side. The shaded area corresponds to the zone containing all the control values obtained in mice not treated with MC. Vertical bars indicate s.e.

that AHH activity was not inducible by polycyclic hydrocarbons in the liver as reported in the literature (Gielen *et al.*, 1972; Nebert & Gielen, 1972). Secondly, we observed that polycyclic hydrocarbons were unable to induce skin AHH activity during either the telogen or anagen phases. On the contrary, when expressed per mg protein, AHH activity was significantly *decreased* on the depilated side as compared to the control side (Fig. 1).

*Skin AHH induction by i.p. methylcholanthrene as a function of the hair cycle*

We studied skin AHH inducibility as a function of the hair growth cycle 24 h after MC injection (Fig. 2). No induction was observed during the first 7 days after depilation. On the 7th day, microscopic examination showed that the hair growth stage varied from mouse to mouse between anagen 3 and 5. Between the 7th and the 11th days, skin AHH inducibility increased abruptly; it peaked around the 11th day and decreased thereafter, disappearing once again after the 18th day.

*Skin AHH induction by topical methylcholanthrene*

MC was topically applied just after depilation and 5, 10 or 21 days later.These mice were then killed the next day. The results (Fig. 3) were very similar to those obtained after i.p. injection; the enzyme was significantly induced in the skin of the animals which had been depilated 10 days earlier and was not modified in the mice depilated 5 or 21 days earlier. Surprisingly, a significant induction was also observed in the group of mice which received the topical MC application the same day as the depilation. The dose–response relationship was also inverted, as previously observed after i.p. MC (Fig. 1). In fact, the dose of 50 μg per mouse was found to be optimal, as shown in Table III.

We have also studied the effect of MC (50 μg/mouse) on another strain of responsive mice (C3H/Ico). In this strain,

TABLE III.—*Effect of topical application of different doses of MC on AHH induction in mouse skin*

| MC administered (μg/mouse) | AHH activity* (% of control) assayed on the 5th day | 10th day |
|---|---|---|
| Control | 100 | 100 |
| 6 | 112 | 161 |
| 12 | 104 | 140 |
| 25 | 121 | 260 |
| 50 | 116 | 292 |
| 100 | 102 | 238 |
| 200 | 108 | 205 |
| 400 | 96 | 164 |

Mice were depilated 5 or 10 days before being killed and were topically treated with MC 24 h before being killed.

* Mean from 3 mice. The control activities were 8·2 and 10·1 pmol/cm² skin/min for mice killed on the 5th and 10th day, respectively.

FIG. 3.—Effect of topical MC application and depilation on skin AHH activity in C57BL/6J mice. Mice were depilated on one side, topically treated with MC (or acetone) on both sides 1, 4, 9 and 20 days later, and killed the next day. Three doses of MC were tested, 50, 150 and 450 $\mu$g per mouse. AHH was measured on both the depilated (+) and non-depilated (−) skin areas. Vertical bars represent the s.e. of the mean of 5 animals. *, significant difference ($P < 0{\cdot}01$) between the results from both sides.

TABLE IV.—*Effect of depilation or shaving and topical MC application on skin AHH activity in C3H/Ico mice*

| Delay (days) | Treatment | AHH activity in Vehicle-treated mice | MC-treated mice |
|---|---|---|---|
| 5 | Shaving | 29·9 ± 5·7 | 30·7 ± 5·5 (N.S.) |
| 5 | Depilation | 31·6 ± 5·1 | 30·5 ± 2·7 (N.S.) |
| 11 | Depilation | 25·7 ± 3·4 | 41·1 ± 4·9 ($P < 0{\cdot}001$) |
| 21 | Depilation | 29·6 ± 6·4 | 29·6 ± 5·7 (N.S.) |

C3H/Ico mice were shaved or depilated 4, 10 and 20 days before the application of the vehicle (acetone) or MC (50 $\mu$g in acetone). They were killed the next day. Each result represents the mean ± s.d. obtained from 5 mice.

skin AHH presents a higher basal activity, but is also specifically induced by MC on the 10th day after depilation (Table IV). In terms of absolute value, the amplitude of induction is about the same as for the C57BL/6J mice, but of course the factor of induction is lower.

Finally, the effect of topical MC application (50 $\mu$g/mouse) was studied in B6, D2 and backcross (D2 × D2B6) mice on the 10th day after depilation, *i.e.* when AHH is most responsive to the treatment. As expected, depilation alone or MC administration to non-depilated mice did not significantly modify AHH activity (Fig. 4). After depilation, topical MC produced a strain-dependent induction. Skin AHH was also induced in a limited number of backcross mice.

If this inducibility follows the same genetic control as described for the other tissues (Gielen *et al.*, 1972) we should have found a clear-cut bimodal distribution of AHH activities. We did not observe two distinct populations, however, but rather a single population in which AHH activity varied from non-inducible to highly inducible levels. A single large population was also observed when the enzyme activities were expressed in u/mg protein. Indeed, one could also consider the possibility of a trimodal distribution: a low (7 mice below 14 pmol/cm$^2$ skin × min), an intermediate (4 mice between 14 and 22 pmol/cm$^2$ skin × min) and a high (12

FIG. 4.—Effect of depilation and topical MC administration on skin AHH induction in DBA/2J, C57BL/6J and backcross mice. The 3 groups of mice were depilated (or not) 9 days before topical administration of MC (or acetone) and killed 24 h later. Each point represents one mouse.

mice over 92 pmol/cm$^2$ skin × min) activity group.

*Comparison of the various methods of expressing the results*

As our enzyme source is a crude skin homogenate, the expression of the results on the basis of protein content was greatly influenced by non-enzymatic proteins which greatly increased after depilation. For this reason, we tried to measure more specific microsomal fraction markers. All proved to be inadequate. Glucose 6-phosphatase (Hers & Van Hoof, 1966) and

TABLE V.—*Comparison of different expressions of AHH activity on the* 10*th day* (*pmol/min*)

| Reference unit | Depilation | Dose of MC (μg) — | 50 | 150 | 450 |
|---|---|---|---|---|---|
| Per cm$^2$ skin | + | 9·6 ± 3·3 | 26·7 ± 1·6 | 18·6 ± 1·8 | 12·9 ± 2·6 |
| | − | 6·9 ± 2·0 | 8·0 ± 1·8 | 5·7 ± 0·7 | 4·5 ± 0·9 |
| | | (N.S.) | ($P$ < 0·001) | ($P$ < 0·001) | ($P$ < 0·001) |
| Per mg protein | + | 4·6 ± 2·2 | 13·4 ± 1·2 | 8·8 ± 0·6 | 6·3 ± 1·4 |
| | − | 5·9 ± 2·8 | 5·8 ± 2·1 | 3·6 ± 0·8 | 3·5 ± 0·9 |
| | | (N.S.) | ($P$ < 0·001) | ($P$ < 0·001) | ($P$ < 0·01) |
| Per mg DNA | + | 51 ± 19 | 137 ± 13 | 97 ± 7 | 63 ± 10 |
| | − | 84 ± 34 | 85 ± 27 | 41 ± 14 | 37 ± 16 |
| | | ($P$ < 0·1) | ($P$ < 0·01) | ($P$ < 0·001) | ($P$ < 0·02) |
| Per nmol EH activity | + | 85 ± 26 | 343 ± 79 | 287 ± 92 | 227 ± 105 |
| | − | 76 ± 20 | 184 ± 25 | 102 ± 29 | 96 ± 73 |
| | | (N.S.) | ($P$ < 0·01) | ($P$ < 0·01) | ($P$ < 0·1) |

The results are expressed as the mean ± s.d. (n = 5) after topical MC (or vehicle) application 9 days after depilation, and killing on the 10th day. The significance of AHH activity differences between depilated and non-depilated areas was obtained by $t$ test.

TABLE VI.—*Comparison of the isotopic and fluorimetric assays for measuring AHH activity in the skin*

| Strain of mice | Days between depilation and killing | AHH activity (pmol/min/cm$^2$ skin) Isotopic Control | Isotopic MC-induced | Fluorimetric Control | Fluorimetric MC-induced |
|---|---|---|---|---|---|
| C57BL/6J | 5 | $6\cdot5 \pm 2\cdot7$ | $7\cdot1 \pm 3\cdot0$ | $0\cdot6 \pm 0\cdot2$ | $3\cdot4 \pm 1\cdot6$* |
| | 11 | $8\cdot0 \pm 1\cdot8$ | $26\cdot7 \pm 1\cdot6$* | $3\cdot1 \pm 1\cdot3$ | $15\cdot7 \pm 6\cdot0$* |
| C3H/Ico | 5 | $31\cdot6 \pm 5\cdot1$ | $30\cdot5 \pm 2\cdot7$ | $3\cdot7 \pm 0\cdot9$ | $8\cdot7 \pm 2\cdot4$* |
| | 11 | $25\cdot7 \pm 3\cdot4$ | $41\cdot1 \pm 4\cdot9$* | $5\cdot9 \pm 1\cdot6$ | $22\cdot3 \pm 6\cdot5$* |
| | 21 | $29\cdot6 \pm 6\cdot4$ | $29\cdot6 \pm 5\cdot7$* | $3\cdot8 \pm 1\cdot5$ | $9\cdot5 \pm 1\cdot9$* |

The methodology is the same as in Table IV, from which some of the data have been drawn.
* $P < 0\cdot001$ compared to the control group (rest not significant).

NADPH cytochrome *c* reductase (Phillips & Langdon, 1962) do not display enough activity in skin, and could not be measured in our samples. The epoxide hydrolase (EH) activity was present at the lower detectable limits and was difficult to measure accurately (Oesch *et al.*, 1977). We also tried to express the results on the basis of the number of cells in the sample, as determined by DNA measurement. Unfortunately, such a marker introduced other difficulties: (a) DNA is not a marker of the enzyme preparation (supernatant of a 1000*g* centrifugation), as it is associated with the nuclei, the subcellular fraction eliminated by centrifugation; a poor homogenization could therefore lower AHH activity without influencing DNA content of the 1000*g* sediment; (b) DNA extraction from the 1000*g* sediment is difficult and losses at this point are hard to avoid.

Table V shows AHH activity in control and MC-treated skin, expressed as a function of different references, *i.e.* skin surface, protein, DNA content or EH activity. These various means of expression do not change the general interpretation of the results, but the statistical significance was diminished when DNA or EH was used as a reference.

*Comparison of results from the isotopic and fluorimetric assays*

As already mentioned, the isotopic AHH measures all the metabolites formed during incubation, whereas the fluorimetric assay, although by far the most used, only takes into account the formation of 3- and 9-hydroxybenzopyrene. Since all the studies on skin AHH performed in other laboratories used the fluorimetric assay, a comparison of the results from both methods was imperative. As shown in Table VI, the two methods produce quite different results. Using the fluorimetric assay, we already observed a clear induction of skin AHH by MC in animals depilated for 5 days and again at 21 days; at the end of the anagen phase (on the 10th day after depilation), the activities are by far higher but the induction factor remains similar. The same phenomenon is observed with both the C57Bl/6J and the C3H/Ico mice.

The discrepancy between the isotopic and fluorimetric assays indicates a great modification in the pattern of the metabolites produced by the skin under the influence of the hair growth cycle. The nature of the metabolites and the extent of conjugation are factors which greatly influence the fluorimetric assay. From a quantitative point of view, the isotopic assay better reflects true AHH activity, but on a qualitative basis the fluorimetric results may also be important. In this respect, the absolute values of AHH activity and the induction factor after MC treatment are both important parameters influenced by the hair growth cycle.

### DISCUSSION

Carcinogenic polycyclic hydrocarbons are normal combustion products of many organic compounds and are important constituents of many types of smoke, such as automobile exhausts, house smoke, various industrial processes and cigarette smoke.

The study of the skin enzymes involved in polycyclic hydrocarbon metabolism is potentially of great interest, as this particular tissue is in direct contact with soot and other combustion products.

Earlier works have demonstrated that skin possesses activating and detoxifying enzymes, notably AHH and epoxide hydrolase (Pannatier *et al.*, 1978; Pyerin & Hecker, 1977). More precise studies have shown that AHH is 4–5 times more active in the epidermis than in the dermis. The highest AHH activity of the dermis occurs in its superficial layer which contains the sebaceous glands and the hair bulb in the telogen phase (Wiebel *et al.*, 1975).

During the hair growth cycle, cell divisions occur throughout the entire skin, from the epidermis down to the hypodermis, where the hair bulbs finally develop. This phenomenon also affects the sebaceous glands, the endothelium cells of the vessels, the wandering cells, and perhaps the fibroblasts too. For this reason we chose to prepare a whole-skin homogenate, rather than separating dermis from epidermis by sophisticated techniques, such as application of a depilatory cream followed by a specific thermal treatment (Slaga *et al.*, 1974). In order to perform a homogenization which would not selectively affect specialized cells, we developed a technique for crushing skin into a powder at liquid $N_2$ temperature. The use of metallic mortar proved to be extremely simple and rapid. In addition, as a result of other liver assays, we showed that freezing in liquid $N_2$ and subsequent thawing do not modify AHH activities, facilitate a subsequent homogenization in a Potter–Elvejhem tube and even produce a slightly more active enzyme preparation.

The goal of this study was to determine whether AHH activity and its inducibility varied during the hair growth cycle and could possibly explain certain variations in skin sensitivity to carcinogens. Indeed, Andreasen & Engelbreth-Holm (1953) demonstrated that 9,10-dimethylbenzanthracene was 5 times more likely to induce skin tumours when it was applied during the resting phase than during the growing phase of the hair cycle. This phenomenon was explained by Berenblum *et al.* (1958) as a result of different carcinogen persistence in the tissues during the resting phase of the hair growth cycle.

Our results show that AHH is only induced by MC during the anagen 5–6 phases. This is contrary to most of the literature, which suggests that skin AHH is induced by MC during the telogen phase (Bouwden *et al.*, 1974; Gelboin *et al.*, 1970; Pannatier *et al.*, 1978; Thompson & Slaga, 1976). As shown in Table VI, this discrepancy arises mainly from the method of assaying AHH. As already mentioned (Van Cantfort *et al.*, 1977) the isotopic method proves to be the best, and the only one producing truly quantitative results, as it takes into account all the metabolites formed under the catalytic action of AHH. On the other hand, the experimental procedure can vary from one laboratory to another. Firstly, following topical MC administration during the telogen phase (Day 0 in our experiments), a significant AHH induction was observed when depilation was performed at the time of MC application. Conversely, no induction occurred when the skin was shaved at the time of killing. One has also to point out that depilation by plucking, as used in our study to induce a synchronized hair growth cycle, is different from spontaneous shedding. Indeed, Silver *et al.* (1969) have demonstrated that increased cellular activity of the epidermis is more pronounced after plucking, which might represent a pathological stimulus similar to a trauma.

When a crude homogenate is used as a source of enzymes, it is always difficult to find an ideal expression for the enzyme activity. As shown in Table V, the statistical significance of the induction phenomenon depends upon the choice of a reference unit. The expression per mg of protein has the advantage of moderating the experimental variations of the enzyme preparation procedures. Nevertheless, the simultaneous extraction of non-microsomal proteins might alter the significance of the results. For example, after MC injection, AHH is significantly lower in the depilated side of the D2 mice (Fig. 1). The expression of the results per unit area of skin seems to be the most reliable, as the results indicate the actual metabolic capacity of the organ, regardless of the number or type of cells present in the sample.

From a toxicological point of view, the fact that skin AHH activity could only be induced by polycyclic hydrocarbons at a certain time during the hair growth cycle might be of prime importance. Skin cells would then have the potential of producing higher amounts of reactive intermediates at the exact time when tissue hyperplasia and consequently promotion mechanisms are very active (Bortwell, 1974). Such a hazardous situation could be even worse if the increased AHH response to induction by the polycyclic hydrocarbon is limited to defined cell types in the skin, a problem at present under study in our laboratory.

This work was supported by Grant 1072 from the Council for Tobacco Research—U.S.A., Inc.

The authors would like to thank Micheline Poma and Jocelyne Doyen for their technical assistance.

The authors are grateful to Marie-Thérèse d'Arripe, Deborah Zelkowitz and Janice Lynn Delaval for their expert help in the preparation of this manuscript.

### REFERENCES

ANDREASEN, E. & ENGELBRETH-HOLM, J. (1953) On the significance of the mouse hair cycle in experimental carcinogenesis. *Acta Pathol. Microbiol. Scand.*, **32**, 165.

BERENBLUM, I., HARAN-CHERA, N. & TRAININ, N. (1958) An experimental analysis of the hair cycle effect in mouse skin carcinogenesis. *Br. J. Cancer*, **12**, 402.

BERENBLUM, I. (1974) *Carcinogenesis as a Biological Problem*. Eds. Neuberger & Tatum. Amsterdam: North-Holland Publish. Co. p. 67.

BORTWELL, R. K. (1974) The function and mechanism of promoters of carcinogenesis. *C.R.C. Crit. Rev. Toxicol.*, **2**, 419.

BOUWDEN, G. T., SLAGA, T. J., SHAPAS, B. G. & BORTWELL, R. K. (1974) The role of aryl hydrocarbon hydroxylase in skin tumor initiation by 7,12-dimethylbenz(a)-anthracene and 1,2,5,6-dibenzanthracene using DNA binding and thymidine-$^{3}$H incorporation into DNA as criteria. *Cancer Res.*, **34**, 2634.

BURTON, K. (1956) Study of the conditions and mechanism of the diphenylamine reaction for the colorimetric estimation of deoxyribonucleic acid. *Biochem. J.*, **62**, 315.

BUTY, S. G., THOMPSON, S. & SLAGA, T. J. (1976) The role of epidermal aryl hydrocarbon hydroxylase in the covalent binding of polycyclic hydrocarbon to DNA and its relationship to tumor initiation. *Biochem. Biophys. Res. Commun.*, **70**, 1102.

DANSETTE, P. & JERINA, D. M. (1974) A facile synthesis of arene oxides at the K-region of polycyclic hydrocarbons. *J. Am. Chem. Soc.*, **96**, 1224.

DEPIERRE, J. W. & ERNSTER, L. (1978) The metabolism of polycyclic hydrocarbons and its relationship to cancer. *Biochim. Biophys. Acta*, **473**, 49.

GELBOIN, H. V., WIEBEL, F. & DIAMOND, L. (1970) Dimethylbenzanthracene tumorigenesis and aryl hydrocarbon hydroxylase in mouse skin: Inhibition by 7,8-benzoflavone. *Science*, **170**, 169.

GIELEN, J. E., GOUJON, F. M. & NEBERT, D. W. (1972) Genetic regulation of aryl hydrocarbon hydroxylase induction. II. Simple Mendelian expression in mouse tissue *in vivo*. *J. Biol. Chem.*, **247**, 1125.

HEIDELBERGER, C. (1975) Chemical carcinogenesis. *Ann. Rev. Biochem.*, **44**, 79.

HERS, H. G. & VAN HOOF, F. (1966) Enzymes of glycogen degradation in biopsy material. *Methods Enzymol.*, **8**, 525.

HOLDER, G., YAGI, H., LEVIN, W., LU, A. Y. H. & JERINA, D. M. (1975) Metabolism of benzo(a)-pyrene. III. An evaluation of the fluorescence assay. *Biochem. Biophys. Res. Commun.*, **65**, 1363.

JERINA, D. M. & DALY, J. W. (1974) Arene oxides: A new aspect of drug metabolism. *Science*, **185**, 573.

MAZEL, P. (1971) General principles and procedures for drug metabolism *in vitro*. In *Fundamentals of Drug Metabolism and Drug Disposition*. Eds La Du *et al.* Baltimore: Williams & Wilkins. p. 527.

MILLER, E. C. & MILLER, J. A. (1976) The metabolism of chemical carcinogens to reactive electrophiles and their possible mechanism of action in carcinogenesis. In *Chemical Carcinogens*. Ed. Searle. Washington, D. C.: ALS Monograph, Vol. 173. p. 737.

MILLER, E. C. (1978) Some current perspectives on chemical carcinogenesis in humans and experimental animals: Presidential address. *Cancer Res.*, **38**, 1479.

MONTAGNA, W. (1962) *The Structure and Function of Skin*. London: Academic Press. p. 247.

NEBERT, D. W. & GIELEN, J. E. (1972) Genetic regulation of aryl hydrocarbon hydroxylase induction in the mouse. *Fed. Proc.*, **31**, 1315.

NEMOTO, N., TAKAYAMA, S. & GELBOIN, H. V. (1978) Sulfate conjugation of benzo(a)pyrene metabolites and derivatives. *Chem.-Biol. Interact.*, **23**, 19.

OESCH, F. (1973) Mammalian epoxide hydrase: Inducible enzymes catalyzing the inactivation of carcinogenic and cytotoxic metabolites derived from aromatic and olefinic compounds. *Xenobiotica*, **3**, 305.

OESCH, F., GLATT, H. & SCHMASSMAN, H. (1977) The apparent ubiquity of epoxide hydratase in rat organs. *Biochem. Pharmacol.*, **26**, 603.

PANNATIER, A., JENNER, P., TESTA, B. & ETTER, J. C. (1978) The skin as a drug metabolizing organ. *Drug Metabolism Rev.*, **8**, 319.

PHILLIPS, A. H. & LANGDON, R. G. (1962) Hepatic triphosphopyridine nucleotide cytochrome c reductase: Isolation, characterization and kinetic studies. *J. Biol. Chem.*, **237**, 2652.

PYERIN, W. G. & HECKER, E. (1977) On the biochemical mechanism of tumorigenesis in mouse skin. VIII. Isolation and characterization of epidermal microsomes and properties of their arylhydrocarbon monooxygenase and epoxide hydratase. *Z. Krebsforsch.*, **90**, 259.

SCHMASSMAN, H. U., GLATT, H. R. & OESCH, F. (1976) A rapid assay for epoxide hydratase activity with benzo(a)pyrene-4,5-(K-region) oxide as substrate. *Anal. Biochem.*, **74**, 94.

SILVER, A. F., CHASE, H. B. & ARSENAULT, C. T. (1969) Early anagen initiated by plucking compared with early spontaneous anagen. In *Advances in Biology of Skin*. Eds. Montagna & Dobson. Oxford: Pergamon Press. p. 265.

SIMS, P. & GROVER, P. L. (1974) Epoxides in polycyclic aromatic hydrocarbon metabolism and carcinogenesis. *Adv. Cancer Res.*, **20**, 165.

SIMS, P., GROVER, P. L., SWAISLAND, A., PAL, K. & HEWER, A. (1975) Metabolic activation of benzo(a)pyrene proceeds via a diol-epoxide. *Nature*, **252**, 326.

SLAGA, T. J., DAS, D. B., RICE, J. M. & THOMPSON, S. (1974) Fractionation of mouse epidermal chromatin components. *J. Invest. Dermatol.*, **63**, 343.

THOMPSON, S. B. A. & SLAGA, T. J. (1976) Mouse epidermal aryl hydrocarbon hydroxylase. *J. Invest. Dermatol.*, **66**, 108.

THORGEIRSSON, S. S. & NEBERT, D. W. (1977) The Ah locus and the metabolism of chemical carcinogens and other foreign compounds. *Adv. Cancer Res.*, **25**, 149.

VAN CANTFORT, J., DE GRAEVE, J. & GIELEN, J. E. (1977) Radioactive assay for aryl hydrocarbon hydroxylase. Improved method and biological importance. *Biochem. Biophys. Res. Commun.*, **79**, 505.

VAN CANTFORT, J., MANIL, L., GIELEN, J. E., GLATT, H. R. & OESCH, F. (1979) A new assay for glutathione S-transferase using [$^3$H]-benzo(a)-pyrene 4,5-oxide as substrate. Inducibility by various chemicals in different rat tissues compared to that of aryl hydrocarbon hydroxylase and epoxide hydratase. *Biochem. Pharmacol.*, **28**, 455.

WEISBURGER, E. K. (1978) Mechanisms of chemical carcinogenesis. *Ann. Rev. Pharmacol.*, **18**, 395.

WIEBEL, F. J., LEUTZ, B. S. & GELBOIN, H. V. (1975) Aryl hydrocarbon (benzo(a)pyrene) hydroxylase: a mixed-function oxygenase in mouse skin. *J. Invest. Dermatol.*, **64**, 184.